# The effect of social media entrepreneurship on sustainable development: Evidence from online clothing shops in Bangladesh

**DOI:** 10.1016/j.heliyon.2023.e19397

**Published:** 2023-08-22

**Authors:** Md Mominur Rahman, Md Jahid Hasan, Bishawjit Chandra Deb, Muhammad Shajib Rahman, Anower Sadath Kabir

**Affiliations:** a Department of Business Administration, Northern University Bangladesh, Dhaka, Bangladesh; b Department of Management Studies, Comilla University, Cumilla, Bangladesh; c Department of Accounting and Information Systems, Comilla University, Cumilla, Bangladesh

**Keywords:** Women empowerment, Social media entrepreneurship, Sustainable development, Family income, Women involvement

## Abstract

The study examines the effect of rural women's participation in social media entrepreneurship on sustainable development in Bangladesh. The study collects 376 responses from the entrepreneurs of online clothing shops employing the simple random sampling technique. The data analysis was conducted using the PLS-SEM technique using Smart PLS 3.3v software. Developing the quantitative research design, the authors test the conceptual model by employing the deductive approach. The study finds a positive effect of women's participation in social media entrepreneurship on sustainable development within online clothing shops in Bangladesh. Bangladesh is capable of creating employment opportunities for rural women through ICT project offerings. Consistently this study also proves social media entrepreneurship increases women's contribution to their family income. Furthermore, this study finds that rural women's family income contribution positively affects sustainable development in Bangladesh. The study can help to achieve SDG 5: Gender Equality and Vision 2041 of Bangladesh at the earliest. Thus, the government, policymakers, and academics can use the study findings as a policy dialogue.

## 1 Introduction

Entrepreneurship plays a crucial role in driving economic development and empowering individuals [1]. The development and meteoric rise of social media in the last few years have radically altered the business landscape by providing brand new channels of product and service promotion for business owners [2]. Among various social media platforms, Facebook, YouTube, and Instagram have gained significant popularity in Bangladesh, where a large portion of the population actively engages in social media activities [1,2]. Emerging phenomena can have far-reaching effects on the corporate world; one such phenomenon is social media, popularly known as Web 2.0 [1]. There is a wide range of social media sites, from closed social networks to public blogs and microblogs. There has been a lot of progress and expansion in the realm of social media [3]. Though My-Space was one of the earliest social networking sites, Facebook, YouTube, and Instagram rapidly overtook My-Space. According to the study, most Bangladeshi adults are on Facebook [4]. Social media users can keep in touch with people they know or meet new people in the virtual world using these platforms. LinkedIn, for example, is a professional networking platform.

Social media platforms have become vital tools for businesses and entrepreneurs when promoting products and services. As a result of social media, their products and services, and more crucially, their visions, have been made public [5]. Consequentially, social media entrepreneurship is often likened to other types of regular media entrepreneurship and used in conjunction with descriptions such as internet entrepreneurs and techno-entrepreneurs. However, social media entrepreneurship must be dealt with differently from other entrepreneurial types. The main reason is that the work is conducted through social media platforms. However, a social media entrepreneur needs to work with infrastructure in internet access and innovation [6]. These structural differences are other factors that distinguish social media entrepreneurs from traditional media. As can be understood from the above, social media entrepreneurs differ from different entrepreneurial types both in terms of the way they do business and the instruments they need, as well as the structural differences they should have. That is why the authors develop conceptual framework to examine the impact of social media entrepreneurship and sustainable development in the context of online clothing shops.

According to Hechavarría and Ingram [7], women are more likely to start businesses in an environment with few constraints, a positive regulatory climate, a streamlined business and legal framework, and a cultural norm that values entrepreneurship. Rahman, Dana [8] investigate the difficulties rural Bangladeshi women confront in maintaining family businesses. According to their research, rural women entrepreneurs in Bangladesh confront significant barriers in the areas of society, culture, money, and training.

It is addressed that social media-based entrepreneurship requires very little capital and minimum infrastructure with basic technical know-how to start. In line with this argument, this region's people's income level, education, and technological know-how are minimal. Thus, social media-based entrepreneurship, particularly Facebook commerce (F-Commerce) become a popular form of business to start a livelihood. Moreover, due to technological advancement in recent years, people even in remote area got the access to the internet and other communication facilities. This facilitates the people from rural and urban Bangladesh to start a new form of business called social media entrepreneurship. Moreover, this form of business opportunity enables women to make their long-cherished shift from traditional economic activities, particularly home-based farming, to a complete business structure [9]. In this business, women simply run a Facebook page to offer, attract, connect and communicate with customers. In this regard, they only need a smartphone/laptop with an internet connection.

Due to the Covid-19 pandemic, many women in Bangladesh working in Small and Medium Enterprises, industrial and commercial establishments have lost their job [10]. Many of these women are now utilizing social media to start their enterprises, albeit this is not a simple feat. In this regard, social media is quickly emerging as one of the most effective resources for women who wish to launch new businesses. This is because it enables them to quickly and easily connect with potential customers, engage in two-way conversations with them, and solicit their opinions and advice on their online goods and services [11]. Social media marketing is also a cost-effective and straightforward method of reaching customers [12].

In recent years, there has been a notable surge in women's involvement on social media platforms within developing nations [13]. The 2020 Mobile Gender Gap Report by the Global System for Mobile Communications (GSMC) unveiled a significant gap: only 16% of women utilize mobile internet, while 33% of men do. In 2019, mobile internet awareness stood at 73% for men and 71% for women [14]. Findings from a distinct study, the Bangladesh National ICT Household Survey 2018–2019, indicated that 34.2% of women respondents are internet users, contrasting with a higher 53.2% among participating men [15].

In the 21st century, social media has brought people together more than ever [16]. It has altered how individuals live their lives and, as a result, has made communication simpler. Social media is the newest medium for enhancing and enriching women's voices [1]. It is the most effective method of raising awareness about women's rights because of its rapidity and broad reach. It has the ability to increase women's independence and decision-making authority, both of which are essential indicators of empowerment. No other industry could have done it as vividly and forcefully as social media has in highlighting the atrocities experienced by women and empowering women in the digital age [17]. By transcending time and space, social media has given women a newfound voice and a greater sense of agency.

Women in rural areas have an essential role in development. They catalyze the economic, environmental, and social transformations necessary for long-term prosperity [18]. However, they confront several difficulties, including restricted access to financing, health care, and education. Encouraging women's empowerment is crucial for the well-being of families, rural communities, and agricultural economies globally since women make up a significant percentage of agricultural workers worldwide. Social media creates a platform for rural women to become entrepreneurs and contribute to society [14].

Research on the social business fund by Ferdousi and Mahmud [15], challenges of women empowerment by Chowdhury and Rabbani [19], microcredit programs contribute to the development of women entrepreneurship by Chowdhury [20], training model for women entrepreneurship program by Idrus, Pauzi [21], factors affect the development of women entrepreneurship [22,23], factors influencing women's empowerment by Tabassum, Begum [24], sustainable development and women status [25,26], women empowerment for sustainable development in India by Lohani and Aburaida [18], were conducted. In Bangladesh, there is no primary data-based research regarding rural women empowerment through social media entrepreneurship and its relation to family income and sustainable development is not yet conducted.

However, Bangladesh signed in the Sustainable Development Goals (SDG) Convention and worked with the help of the United Nations (UN) to address the major development challenges faced by the people here. The endeavour is in line with the agenda of the UN to be achieved by 2030. Among the 17 SDGs, achieving gender equality is at number 05, which is crucial for Bangladesh to perform where the number of women is slite more than men in the total population. The men-to-women ratio is 95:100, while the global average is 105 men to 100 women (Gender Ratio, 2022). Without ensuring women's participation in economic activities, gender equality is never possible.

The government of Bangladesh has a strong incentive to prioritize the Sustainable Development Goals (SDGs) due to their potential to address major development challenges and improve the well-being of its citizens [27]. By aligning with the SDGs, Bangladesh demonstrates its commitment to sustainable and inclusive growth [8]. The SDGs provide a comprehensive framework that guides the government's efforts to eradicate poverty, ensure gender equality, promote education, combat climate change, and achieve overall sustainable development [1,28]. Prioritizing the SDGs can also attract international support and foreign investments, contributing to a more prosperous and equitable future for Bangladesh [29]. Focusing on women in entrepreneurship is essential for promoting gender equality and women's empowerment, which are fundamental human rights and key components of the SDGs [7]. Women's entrepreneurship has the potential to drive economic growth, job creation, and innovation [24]. By enabling women to participate in economic activities and access financial resources, societies can break down gender barriers and create more inclusive and equitable societies [30]. Additionally, women's entrepreneurship can contribute to poverty alleviation, financial independence, and overall well-being, particularly for marginalized women in rural areas [31].

Women play a critical role in achieving the SDGs due to their multifaceted contributions to society. Their participation in decision-making processes is crucial for effective governance and policy formulation [32]. Women's involvement in various sectors, such as education, health, agriculture, and environmental conservation, can lead to improved access to services, enhanced food security, and sustainable resource management [33]. Gender equality, as outlined in SDG 5, is recognized as a key enabler for the attainment of all other SDGs [34]. When women are empowered and have equal opportunities, societies benefit from their knowledge, skills, and contributions, leading to more prosperous and resilient communities [24]. Examining the intersection between female entrepreneurs, online platforms, and the digital sphere Understanding the complexities of today's business practices requires an appreciation for the importance of entrepreneurship. These days, it's impossible to imagine doing company without using social media [3]. By harnessing the potential of social media entrepreneurship, women can overcome traditional barriers to entry and establish successful online businesses (Hossain and Rahman, 2018). Social media entrepreneurship offers flexibility and can be conducted from anywhere with minimal infrastructure requirements, making it particularly beneficial for women in rural areas [35]. Additionally, studying this relationship provides insights into innovative business models, the role of technology in economic empowerment, and the potential for inclusive and sustainable economic growth [17].

Moreover, the Bangladesh government has already announced the 2021–2041 Perspective Plan (PP2041), which is mainly known as Vission 2041, that aims to get rid of extreme poverty, graduate to the Upper Middle Class by 2030, and achieve the status of a high economic nation by 2041 [29,36]. So, the active participation of both men and women in financial and other development activities is necessary. However, the employment opportunity in Bangladesh is minimal since the number of people flowing into the job market every year is several times greater than the vacant position. Moreover, covid-19 pandemic accelerated the unemployment rates, as reported by ILO 2022. Bangladesh is embracing a 0.6% higher unemployment rate, and 3.6 million people are expected to be unemployed in 2022. In this regard, doing business online using social media is a prospective option that requires very little capital and other arrangements to start a business. Thus, this study is relevant to Bangladesh's national agenda to achieve SDG-05 and Vision 2041. To fill the gap in the past studies, the researchers seek the answer to the question, “What are the effects of women's participation in social media entrepreneurship on sustainable development in Bangladesh?” Thus, this study examines the effect of rural women's participation in social media entrepreneurship on sustainable development in Bangladesh. Further, the study specifies the following objectives: to explore the relationship between women's participation in social media entrepreneurship and rural women's family income; to explore the relationship between rural women's family income and sustainable development; and finally, to examine the relationship between women's participation in social media entrepreneurship and sustainable development.

## 2 Literature review and hypothesis formation

### 2.1 Social media entrepreneurship and rural women's family income

It is assumed that social media entrepreneurship plays a crucial role in building and promoting online clothing shops and stimulating economic activity. Social media is widely accepted to create an entrepreneurship framework where women can contribute to family income and national development. Due to the expansion of digitalization in business, now rural women can contribute to family income like urban women with the development of social media. Some studies support this by Nukpezah and Blankson [37]; social media create an excellent opportunity to do business for both men and women, especially for women who live in rural areas. They can buy and sell products by using social media accounts. Proponents of social media have agreed that social media enhance women's empowerment [9]. Now, rural women are not lagging behind urban women. Thus, they can easily contribute to the family income through the income they earn through doing business on social media. As per Gavino, Williams [35], for women entrepreneurs, social media is a powerful tool for advertising, marketing, and attracting customers, ultimately leading to sales. Therefore, a woman from a rural area can contribute to the family income and boost women's empowerment in society [38].

Evidence from Oman [9,30] pointed out barriers faced by rural women entrepreneurs in Oman, India [28,39] highlighted that rural women's entrepreneurial development improves their capacities and decision-making status in the family and society, Iraq [40], shows potentials for rural women's entrepreneurship and other studies in developed countries [31,41], address the women entrepreneurship and economic development. However, several studies [1,6,42] found that social media creates entrepreneurs or is linked with entrepreneurship. Even though social media entrepreneurship as a critical force of women's entrepreneurship associated with rural women's family income has been pointed out recently, no single study incorporates that social media entrepreneurship enhances rural women's family income [31]. The emergence of social media has unquestionably provided women with fresh entryways to previously unattainable opportunities in Bangladesh. In Bangladesh, the landscape of social media-driven female entrepreneurship is expanding. This evolution can be attributed to the concurrent rise of Western-oriented economic reforms that are carving out avenues for women to embark on, influence, and oversee their own business ventures. Despite this progression, a notable gap remains: there exists no singular study that comprehensively illustrates the impact of social media entrepreneurship on the income of rural women's households in Bangladesh. In Bangladesh, women and men are almost in the same proportion compared to men. Bernzen, Jenkins [43] state that employment opportunity, especially for women, is not significant in Bangladesh to develop women's entrepreneurship. In recent years, many firms have set up shop on social networking platforms (or social media). Social media entrepreneurship creates employment opportunities for rural women to become successful entrepreneurs [1]. Based on this argument, the authors develop the following hypothesis;

H1 *A significant positive relationship exists between women's participation in social media entrepreneurship and rural women's family income.*

### 2.2 Social media entrepreneurship and sustainable development

The realm of social media has brought about a revolutionary transformation in how we communicate, collaborate, consume, and create. Within this dynamic context, the emergence of social media entrepreneurship stands as a vital catalyst for nurturing female entrepreneurs. Its potential influence on entrepreneurial progress in societies, leading to sustainable development, is both profound and expected. As contemporary society increasingly recognizes entrepreneurship as a viable economic avenue, the realm of social media has paved the way for the ascent of entrepreneurs, particularly among women [6]. The role played by social media entrepreneurship is substantial, not only in advancing women's empowerment but also in fostering economic growth. The rapid expansion of social media in Bangladesh has prompted scholars to advocate for an exploration of its impact on entrepreneurship and economic development [44]. A transformation in women's aspirations toward economic and social self-sufficiency, compared to past generations, has become evident, encouraging more women to embark on entrepreneurial journeys, as noted by Lohani and Aburaida [18]. In a societal landscape marked by prevalent gender and class stereotypes, witnessing women surmount socio-economic obstacles and establish their enterprises through the leverage of social media serves as an inspiring narrative. While entrepreneurial activities were traditionally clustered in urban centers, the realm of social media entrepreneurship has birthed a new cohort of rural women entrepreneurs, a phenomenon catalyzed by its growth and expansion.

Most women entrepreneurs were involved in producing personal wear (27.8%), knitwear and ready-made clothes (12.9%), and agro-processing and agribusiness (10.8%), according to research by the SME Foundation in 2009 (Ghosh, Ghosh [45]). About 46.8% of the women entrepreneurs were involved in various trades like tailoring, handicraft production, beauty parlour printing and fitness centers, culinary services, and confectionery. Social media entrepreneurship changes the pattern of women's empowerment [14] argues that it creates an employment platform for other women and helps them become successful entrepreneurs.

To achieve sustainable development goals, social media entrepreneurship must play a key role in gender equality, reducing poverty, reducing inequalities, creating jobs, and supporting underprivileged groups, such as women and minorities, such as youngsters. Women comprise 34% of Bangladesh's workforce, and a 10% increase in female engagement in labor is predicted to increase GDP growth by 1% [46]. Consequently, women's entrepreneurial efforts have become a major driving force in the country's economic and social development, which is required to achieve SDG. Bangladesh's number of women entrepreneurs has increased by roughly 90% in the last few years. The majority are due to social media entrepreneurship engagement [47]. In addition, rural women can easily trade their products through social media platforms. Olanrewaju, Hossain [1] argue that social media entrepreneurship greatly enhances economic growth, ensuring sustainable development by creating employment opportunities and removing poverty. This study argued that social media entrepreneurship could help in achieving SDG 5: Gender Equality, SDG-1: No Poverty, and SDG-10: Reduced Inequalities through creating women empowerment.

The insights of Surugiu and Surugiu [48] converge in recognizing the contribution of social media entrepreneurship to the advancement of sustainable development. Previous researchers have also demonstrated the correlation between entrepreneurship and engagement with social networks, illustrating its impact on economic growth, particularly in the context of China [49]. Nevertheless, the landscape is nuanced, as evidenced by divergent outcomes observed within a singular study that encompassed diverse religious backgrounds [49,50], highlighting that the relationship isn't universally straightforward. Despite this complexity, the comprehensive exploration of the interplay between social media entrepreneurship and sustainable development remains an area with limited scholarly attention. This gap is notable considering the potential significance of such research in the broader realm of women's empowerment. Consequently, the nexus between social media entrepreneurship and the realization of Sustainable Development Goals (SDGs) becomes notably intertwined. Although a handful of studies have ventured into the realm of social media entrepreneurship's role in structuring sustainable development [29,44], the intricate threads binding the two warrant a more profound investigation and discourse. Framed within this rationale, the authors posit the following hypothesis:

H2 *A significant positive relationship exists between women's participation in social media entrepreneurship and sustainable development.*

### 2.3 Rural women's family income and sustainable development

Elevating the status of women stands as a paramount objective during the ongoing pre-accession processes in both developed nations and developing counterparts like Bangladesh. This imperative underscores the need to discern the obstacles encountered by women and their distinct preferences, thereby propelling the advancement of women's roles within society, particularly among rural communities. Undoubtedly, the economic wellbeing of rural women's households serves as a linchpin for establishing gender parity and minimizing disparities, thereby fostering enduring progress within advanced economies. Furthermore, the disparities of economics and societal standing between genders are markedly more pronounced in rural landscapes [33]. The drive for gender equality and the empowerment of women have emerged as pivotal elements in the pursuit of the Millennium Development Goals since 2000. Compelled by these imperatives for development, governments and pivotal national policymakers are increasingly heeding the empirical insights documented by the United Nations Development Program. These insights illuminate the intrinsic link between the familial incomes of rural women and the augmentation of sustainable development [51], catalyzing a more earnest consideration of this dynamic association.

Ghosh, Ghosh [45] argues that a family's income is affected by social media entrepreneurship. New mindsets exist in an era of increasing income disparity, unemployment, and global warming [52]. Increasing family income through social media business contributes to sustainable development. According to Franceschelli, Santoro [53], the social media business is a realistic and significant option for achieving sustainable development. Women's employment increases income contribution to the family [54]. Social entrepreneurship influences the economic growth of a local area. It has become increasingly common in established and emerging nations for entrepreneurship to be the sole source of employment and industrial growth. Women's entrepreneurship is a more recent phenomenon in most economies. Investing in the family's well-being has a favorable impact on a wide variety of SDGs [27]. A good example of this is the link between addressing poverty and multiple deprivations and achieving some of the Sustainable Development Goals (SDGs) by making it easier for families to meet their personal subsistence goals, such as access to health care, education, and clean water, as well as a broader range of educational and employment opportunities. As per Hechavarría and Ingram [7], entrepreneurship development can enhance the socio-economic situation of underdeveloped countries. With the expansion of family income, national growth is greatly impacted and enhances overall sustainable development in a country. As far as the authors are aware, there exists no study that investigates the correlation between the improved family income of rural women and the advancement of sustainable development. This gap is not exclusive to Bangladesh; it extends to analogous economies as well [55].

H3 *A significant positive relationship exists between rural women's family income and sustainable development.*

Further, this research employs the feminist theory of development that argues that gender inequality is a root cause of poverty and undermines sustainable development [32,56]. The feminist theory of development recognizes that gender equality is an essential component of sustainable development and that patriarchal norms and gender-based discrimination often prevent women from fully participating in the economy and accessing resources and opportunities [57]. This theory highlights the need to address the systemic and institutional barriers that limit women's agency, including unequal access to education, health care, and credit, as well as discriminatory laws and social norms restricting women's ability to own property, inherit, or control themselves. According to this theory, gender equality is not only a human rights issue but also a development issue. It can increase economic growth, reduce poverty, and promote sustainable development [57,58]. By addressing gender inequalities and promoting women's empowerment, it is possible to increase women's productivity and economic contributions and reduce poverty and inequality.

**Fig. 1:**
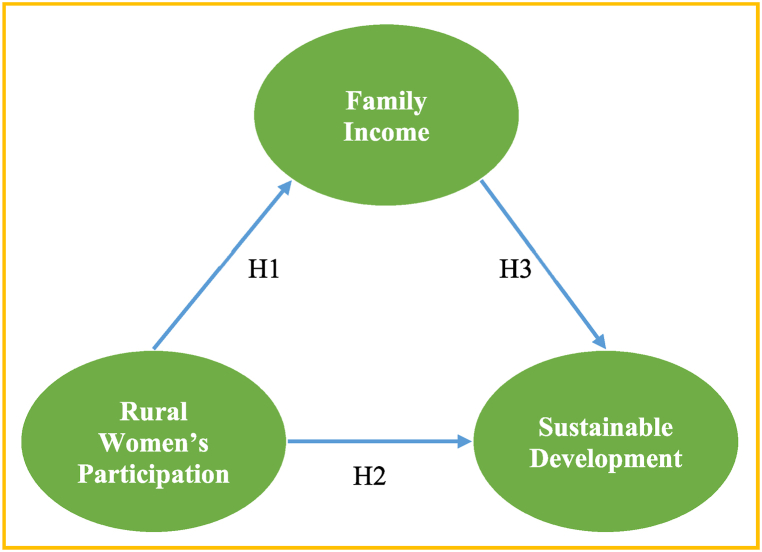
Conceptual framework of the study.

The authors developed the following conceptual model based on the literature survey and theoretical underpinning (see Fig. 1).

## 3 Methods

The study employs a quantitative research methodology as the quantitative research approach is easily verifiable and scientific [59]. Positivism is a phrase that refers to a method of studying society that is based entirely on scientific data, such as experiments and statistics, to ascertain the fundamental nature of how society functions [60]. The study, thus, applies the positivism approach to find the effect of social media entrepreneurship on sustainable development. Through deduction reasoning, the researchers tested the hypothesis on three dimensions. First, the researchers tested whether women's empowerment through social media entrepreneurship affects sustainable development. Second, it tested whether family income is affected by social media entrepreneurship. Finally, the researchers deduced whether family income affects sustainable development.

To test the research questions, the researchers collect data from clothing shops over online media. The researchers ensured the rural women through their rural residential status. The researchers asked why these online clothing shops contributed earnings to their family income. The respondents mentioned that their contributions were mandatory to conduct the family. The respondents also mentioned that their clothing products were more highly available to diverse locations and buyers than physical shops. The researchers use Google Forms as the data collection tool. This research got informed consent from all of the participants before proceeding with data collection. The study collects a list of 680 online clothing shops and makes a random number using Randon Number Generator (www.random.org). The researchers send the questionnaire to 680 respondents through inbox-SMS following the random number lists. The researchers got 376 responses back and analyzed those data. Before sending the questionnaires to the respondents, the researchers did a pre-testing by the three academicians. The researchers adjusted the suggestions taken from the piloting and pre-testing, as Easterby-Smith, Jaspersen [61] suggested. The questionnaire had two sections, including the profile of the participants and the statements. The researchers employ the simple random sampling method in collecting the data following Hair, Page [62]. For the randomization, the researchers collect the profile name of the online clothing shops numbering cardinally, then apply the cardinal number to www.random.org to generate the random number automatically. By getting the random number, the researchers reach the participants accordingly. More reliable, smoother to use, and a better representation of a respondent's objective opinion has been found when using seven-point Likert scale items [60,63]. 7-point items are the best option for surveys like those used in usability evaluations, especially compared to higher-order items [60,64]. Thus, the questionnaire has been designed based on a seven-point Likert scale in this study.

This research selects the clothing shops sector in Bangladesh based on several factors. Firstly, the clothing shops sector typically requires lower capital investment than other sectors such as manufacturing or construction. This makes it a more accessible option for rural women entrepreneurs with limited financial resources to start a business. Secondly, the ready-made garment industry significantly contributes to Bangladesh's economy, and many designs and styles are available for female entrepreneurs to source from. This means that rural women entrepreneurs can tap into the existing supply chain and benefit from economies of scale, making it easier to start a business and offer competitive prices. Lastly, the clothing shops sector is generally more manageable for women looking to start a small business. In Bangladesh, women's involvement in the formal labor force remains modest, leading the clothing retail sector to emerge as a feasible avenue for women to secure earnings and contribute to their families' upkeep without compromising their domestic obligations.

The study employs the PLS-SEM methodology to scrutinize the collected data. Hair, Hult [65], Rahman and Akhter [66], and Shmueli, Sarstedt [67] stated that PLS-SEM offers the evaluation of multiple cause-effect associations in path models containing latent constructs. The path model is appropriate as the study tests multiple relationships (Hair et al., 2012); PLS-SEM can be chosen.

The researchers adopt the indicators of the constructs (Social Media Entrepreneurship, Family Income, and Sustainable Development) from past research studies [1,68]. The indicators of social media entrepreneurship through women have been adopted from Khajeheian [68], Hossain [17], and Olanrewaju, Hossain [1]. The researchers took the family income construct's indicator from Maître, Whelan [54], Starbird, Norton [34], and Roy, Haque [69]. Finally, the indicators of sustainable development were adopted from Lohani and Aburaida [18], Tabassum, Begum [24], and Akhter and Cheng [70].

The first construct, Rural Women's Participation (RWP), includes five items that measure the extent to which the respondents agree that rural women are actively initiating social businesses on online platforms (RWP1), rural women's participation in social businesses has increased due to the COVID-19 pandemic (RWP2), rural women are using social media platforms to contribute to social businesses (RWP3), rural women play a critical role in supporting their households and communities in achieving food and nutrition security through social businesses (RWP4), and rural women's participation in social businesses is an important source of rural economic growth (RWP5). The second construct, Family Income (FIN), includes four items that measure the extent to which the respondents agree that rural women are generating income to contribute to their family's needs through social businesses (FIN1), rural women are successfully improving rural livelihoods and overall well-being through family income from social businesses (FIN2), the desire for better economic opportunities for their families motivates rural women to pursue a career in social media entrepreneurship (FIN3), and rural women are contributing a significant portion of their family's expenditures through income from their social businesses (FIN4).

The third construct, Sustainable Development (SD), includes five items that measure the extent to which the respondents agree that rural women's participation in social media entrepreneurship is contributing to gender equality (SD1), rural women's participation in social businesses is contributing to alleviating poverty in the rural economy (SD2), rural women are supporting the overall economic growth of the country through their social media entrepreneurship (SD3), rural women's participation in social businesses is improving the family income status, resulting in better standards of living (SD4), and rural women's involvement in social media entrepreneurship is contributing to sustainable economic and social development (SD5). The study examines how these constructs are related and finds that rural women's participation in social media entrepreneurship positively affects sustainable development in Bangladesh, as it contributes to gender equality, alleviates poverty, supports economic growth, and improves family income status. The study also finds that rural women's family income contribution positively affects sustainable development in Bangladesh, indicating that social media entrepreneurship can be a powerful tool for achieving sustainable development goals in rural communities.

## 4 Results

### 4.1 Results of measurement model

**Fig. 2:**
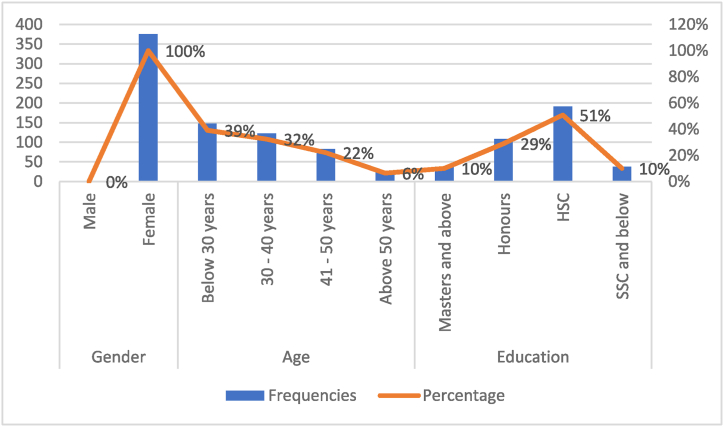
Profile of the participants developed from the collected data.

Within Table 1, the analysis of the measurement model unfolds, encompassing critical indicators such as the mean, standard deviation, factor loadings, as well as statistical measures including Cronbach's alpha (α), rho, composite reliability (CR), and the average variance extracted (AVE). Simultaneously, Fig. 2 captures the respondents' demographic panorama, encompassing factors such as gender, age, and educational attainment. Notably, the questionnaires received a full completion rate from female participants, constituting 100%, while male participants exhibited a 0% completion rate, collectively manifesting their keen engagement with the study's objectives. Regarding age, 39% of participants were below 30, 32% were from 30 to 40, and 6% were above 50. Thus, it is noticeable that most participants were 40 years and below. Further, respondents were asked about their education level. Most of the respondents completed HSC (higher secondary certificate). Additionally, SSC in Fig. 2 indicates the completion of a secondary school certificate.

**Table 1:** Measurement model analysis.

Constructs	Items	FL***	α	rho	CR	AVE
Rural Women's Participation (RWP)	RWP1	0.715	0.800	0.808	0.861	0.555
	RWP2	0.743				
	RWP3	0.684				
	RWP4	0.856				
	RWP5	0.715				
Family Income (FIN)	FIN1	0.710	0.805	0.813	0.873	0.633
	FIN2	0.815				
	FIN3	0.796				
	FIN4	0.854				
Sustainable Development (SD)	SD1	0.798	0.824	0.831	0.877	0.588
	SD2	0.799				
	SD3	0.743				
	SD4	0.795				
	SD5	0.693				

Abbreviations: FL = Factor Loading, CR= Composite Reliability, AVE = Average Variance Extracted, α = Cronbach's alpha. ***All indicators are significant at p < 0.001.

In order to ascertain the robustness of construct reliability and validity, the study undertook a comprehensive measurement model analysis, as outlined in Table 1. The results highlighted noteworthy indicators: Cronbach's alpha coefficients surpassing the threshold of 0.80, in alignment with the guidance of Hair, Hult [65], ensuring the constructs' reliability and internal consistency. Significantly, the researchers adhered to the counsel of Hair, Hult [65], Shmueli, Sarstedt [67], and Sarstedt, Ringle [71], reflecting their commitment to methodological rigor. Impressively, the reported rho values for each construct consistently exceeded 0.80, further affirming their reliability. In tandem with these examinations, the internal consistency of the measurement scales was evaluated through the lens of composite reliability (CR). Demonstrating unwavering dedication to meticulousness, the CR values for the scales manifested as follows: 0.861 for RWP (rural women's participant), 0.873 for FIN (family income), and 0.877 for SD (sustainable development), echoing the guidelines advocated by Hair, Hult [65] and Shmueli, Sarstedt [67]. This comprehensive approach reinforces the study's meticulous validation and ensures the soundness of the employed constructs.

As emphasized by Saunders, Lewis [60] and corroborated by Sarstedt, Ringle [72], the concept of Average Variance Extracted (AVE) serves as a measure that encapsulates the extent of variation captured by a construct relative to the variance stemming from measurement errors. To establish robust convergence, it is strongly recommended that an AVE of at least 0.50 be attained [60,65,[72], [73], [74]]. Notably, as articulated by Hair, Hult [65], an AVE below 0.50 signifies that survey items contribute more to errors than to the variance within the constructs. Hence, it remains imperative, as outlined by Hair, Hult [65] and Sarstedt, Ringle [72], that each construct within a measurement model undergoes an AVE determination, ensuring a minimum threshold of 0.50 [65,67,75,76]. In the context of this study, it is worth highlighting that all constructs surpass the 0.55 mark for AVE values, substantiated by the data presented in Table 1. This comprehensive adherence to AVE guidelines reaffirms the meticulousness applied in ensuring the validity and robustness of the constructs under scrutiny.

The significance of discriminant validity, as outlined by Shmueli, Sarstedt [67] and underscored by Deb, Rahman [77], pertains to the statistical distinction between two latent variables that represent distinct theoretical concepts, a crucial consideration within PLS-SEM path analysis. Insights gleaned from Table 2, Table 3 underscore the successful establishment of discriminant validity, satisfying the rigorous criteria set forth by the Fornell-Larcker Criterion, Heterotrait-Monotrait Ratio (HTMT), and cross-loadings [63]. Echoing the principles laid down by Fornell and Larcker [78], the squared correlations among latent constructs neatly align between the squared root of AVE, as meticulously demonstrated in Table 2. Embracing the perspective of Shmueli, Sarstedt [67], and Hair, Hult [65], it becomes apparent that HTMT serves as a metric for assessing the resemblance between two latent variables. A critical threshold demands that HTMT be less than one to validate discriminant validity. Remarkably, within the context of this study, the HTMT value comfortably adheres to this benchmark, reaffirming the established discriminant validity.

**Table 2:** Fornell-Larcker criterion and HTMT.

	Family Income	Rural Women's Participation	Sustainable Development
Family Income	***0.796***	0.713	0.690
Rural Women's Participation	0.573	***0.745***	0.843
Sustainable Development	0.565	0.694	***0.767***

Note: The entries along the diagonal correspond to the square root of AVE values. Below this diagonal line, squared correlations are displayed, whereas above it, HTMT measures are presented.

**Table 3:** Cross loadings.

	Family Income	Rural Women's Participation	Sustainable Development
**FIN1**	***0.710***	0.389	0.406
**FIN2**	***0.815***	0.506	0.420
**FIN3**	***0.796***	0.410	0.488
**FIN4**	***0.854***	0.509	0.482
**RWP1**	0.459	***0.715***	0.471
**RWP2**	0.392	***0.743***	0.509
**RWP3**	0.415	***0.684***	0.390
**RWP4**	0.504	***0.856***	0.598
**RWP5**	0.360	***0.715***	0.593
**SD1**	0.350	0.576	***0.798***
**SD2**	0.494	0.622	***0.799***
**SD3**	0.358	0.473	***0.743***
**SD4**	0.504	0.505	***0.795***
**SD5**	0.446	0.463	***0.693***

### 4.2 Model fitness

The meticulous evaluation of model fitness encompasses various critical metrics: f square, R square, adjusted R square, Standardized Root Mean Square Residual (SRMR), Normed Fit Index (NFI), and Root Mean Square Error of Approximation (RMSEA). As articulated by Sarstedt, Ringle [72], the significance of f square lies in its ability to effectively expound the variance accounted for by each independent variable within equations. A compelling assertion is made: f-square effect sizes should exceed 0.35, indicative of substantial effects. Remarkably, within this study, the f-square values boldly surpass the threshold, resonating at 0.429 or higher. This robustness underscores the capacity of exogenous variables to notably explain endogenous variables with remarkable impact.

Turning to the assessment of the path model's fit, the indispensable R-squared statistic takes center stage, aligned with insights from existing scholars [63,65,67]. Positioned as a gauge of data proximity to the fitted path line, R-squared aptly quantifies the extent to which independent variables illuminate variance in dependent variables [67]. A compelling guideline put forth by Hair, Hult [65] necessitates an R square value closer to 1, with a threshold of 0.25 for substantial influence explanation within the path model. In this study's context, the R square values stand at 0.328 and 0.523, exemplifying a robust connection between variables. The indispensable notion of adjusted R square, as outlined by Shmueli, Sarstedt [67], aligns with the researchers' comprehensive approach, accounting for multiple independent variables within a regression model. This pragmatic consideration bolsters the model's rigor and is reflected in the reported results.

Delving into further validation, the indices of SRMR, NFI, and RMSEA are diligently presented, consistent with previous research tenets [63,[65], [66], [67],^72^,^75^,^79^]. The stringent criteria placed down by Hair, Hult [65], Shmueli, Sarstedt [67], and Sarstedt, Ringle [72] delineate that SRMR values should remain under 0.08, RMSEA values should reside beneath 0.06, and NFI values should surpass 0.80. Crucially, Table 4's revelations reaffirm these benchmarks, portraying SRMR values below 0.08, RMSEA values below 0.06, and NFI values surpassing 0.80. Consequently, the model is not just fitting; it's optimally aligned with the data, underpinning the study's robustness and reliability.

**Table 4:** Model fitness.

Variables	f square	R square	Adj. R square	SRMR	NFI	RMSEA
Rural Women's Participation						
Family Income	0.489	0.328	0.327	0.075	0.824	0.049
Sustainable Development	0.429–0.670	0.523	0.521	0.075	0.824	0.049

### 4.3 Path model

**Fig. 3:**
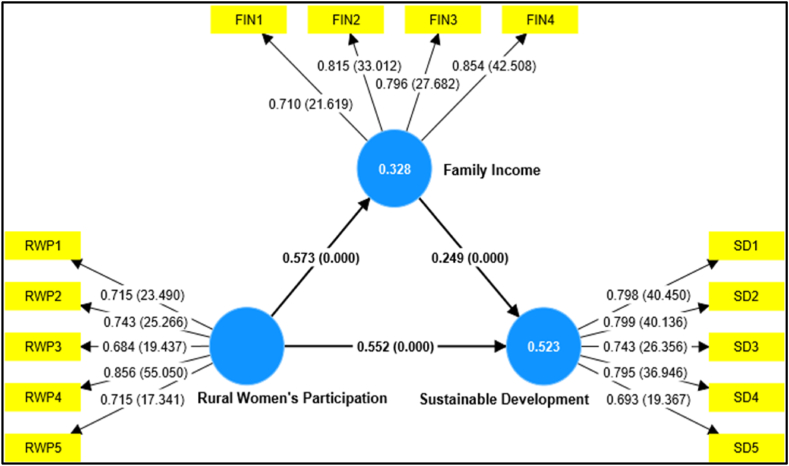
The structural model derived from Smart PLS output.

Illustrated in Table 5 and Fig. 3, the comprehensive path analysis and structural model findings cast a spotlight on pivotal relationships. Notably, the investigation underscores the affirmative link between social media entrepreneurship and women's empowerment (RWP), which in turn yields a positive impact on family income contribution. This significant correlation emerges with striking clarity, boasting a remarkable level of significance below 0.001. The beta coefficient for the RWP and FIN (Rural Women's Participation → Family Income) nexus stands at an impressive 0.573, while the corresponding t-value reaches an astounding 14.690. The robustness of these results unequivocally supports Hypothesis 1 (H1), effectively affirming that heightened engagement in social media entrepreneurship by women directly corresponds to enhanced family income contributions. The implication is profound: the surge in social media entrepreneurship predicts a noteworthy upswing in the economic contributions made by women within their families.

**Table 5:** Analysis of path.

Relationship	Coeff. (β)	t-value	p-values	2.5% CI	97.5% CI	VIF	Decision
Rural Women's Participation **→** Family Income	0.573	14.690	0.000***	0.495	0.648	1.000	H1 supported
Rural Women's Participation **→** Sustainable Development	0.552	12.422	0.000***	0.463	0.639	1.489	H2 supported
Family Income **→** Sustainable Development	0.249	5.002	0.000***	0.151	0.347	1.489	H3 supported

*** <1%. RWP stands for Rural Women's Participation, FIN for Family Income, SD for Sustainable Development, and CI for Confidence Intervals.

Moving forward, the exploration into the relationship between RWP and SD (Rural Women's Participation → Sustainable Development) uncovers another compelling narrative. With a beta coefficient of 0.552 and a t-value of 12.422, this correlation accentuates the transformative potential of increased women's involvement in social media entrepreneurship, correlating with heightened sustainable development outcomes. In essence, Hypothesis 2 (H2) garners robust substantiation, emphasizing that a surge in women's empowerment through social media entrepreneurship directly contributes to an elevated trajectory of sustainable development. The implication reverberates: advancing women's engagement in this domain emerges as a potent driver of overall sustainable progress.

Lastly, the study delves into the interplay between family income contribution and sustainable development. The beta coefficient for the FIN and SD (Family Income → Sustainable Development) connection registers at 0.249, with an accompanying t-value of 5.002. This statistically significant outcome, observable at a level below 0.001, lends unassailable support to Hypothesis 3 (H3). This underscores that augmenting family income contributions directly fosters a positive impact on the trajectory of sustainable development. The implication is clear: bolstering family income through avenues like social media entrepreneurship carries the potential to bolster and steer sustainable development efforts. The findings of this study not only illuminate the interconnectedness of social media entrepreneurship, women's empowerment, family income contribution, and sustainable development but also underline their potential transformative implications across social, economic, and developmental dimensions.

## 5 Discussions

Rural women's empowerment is about boosting women's self-worth, their capacity to make their own choices, and their right to influence societal change for their benefit and the benefit of others. It is inextricably linked to a fundamental human right, which is necessary to establish a more peaceful and prosperous world. Empowering women is critical for a family's, community's, and country's health and social development [45]. Women can attain their most tremendous potential in a safe, joyful, and productive environment. They may contribute their abilities to the workforce and help their children grow happier and healthier. They also contribute to fostering sustainable economies and benefit society and humanity [7].

*Firstly,* we found that women's participation in social media entrepreneurship positively impacts rural women's family income (p < 0.05). It indicates that social media entrepreneurship enhances rural women's family income, which ultimately results in women's empowerment. Thus, through women's participation in social media entrepreneurship, a woman from a rural area can contribute to her family's income and the country's economic development. The results of our research are transferable to other economies with features analogous to Bangladesh's. This research results go in the same direction as previous studies [1]. The rise of social media has given women a new venue for communication, self-expression, political organizing, and activism [18]. Women worldwide have linked via social media and now support one another as professionals, legislators, politicians, and company owners [24]. There's no denying that social media has opened up opportunities and empowered women in Bangladesh. This helps women be autonomous and confident in their decision-making. Empowering women is giving them a voice in political, economic, and social matters [18].

*Secondly*, we found a significant positive association between women's participation in social media entrepreneurship and sustainable development (p < 0.05). It is addressed that women's involvement in social media entrepreneurship creates a framework to achieve sustainable development in line with SDG 05- gender equality and SDG -01 no poverty, and SDG-10: Reduced Inequalities through creating women empowerment. It is stated that SDG 05 and SDG 10 advocate the increased use of technology, mainly information and communication technology (ICT), intending to assist women and girls worldwide in realizing their full potential. In addition, the government has launched a program to support the development of rural women entrepreneurs and established a project to encourage the growth of women entrepreneurship in the ICT sector [70]. The findings of the study are supported by previous studies [29]. The purpose of social media is to provide a forum for people to express themselves online by talking about the things that are important to them. Expressing oneself freely is a key to personal success and contentment. While exposing them to discrimination and cultural pressure, social media also helps them maintain their integrity, confidence, and independence [1]. Rural women's participation is necessary to attain Sustainable Development Goals (SDG) 5 that all kinds of discrimination against all women and girls be eliminated, as well as guaranteeing women's full and practical involvement in decision-making at all political, economic, and public life. SDG 5 also advocates increasing the use of technology, mainly information and communication technology (ICT), to assist women and girls worldwide in realizing their full potential. The ICT department has launched a program called the “She Power Project: Sustainable Development for Women through ICT” with the goal of enhancing the lives of 10,500 females. Freelancers-to-entrepreneurs, IT service providers, and call center representatives are the focus of the project's three corresponding programs. Thus, H1 is supported through this study, indicating the social media entrepreneurship of rural women affects sustainable development.

*Lastly*, there appears to be a significant positive relationship between rural women's family income and sustainable development (p < 0.05). Social media entrepreneurship opens a horizon to generate income, particularly for women. And this added income the rural women contribute to sustainable development. Over time, social media has altered the way women are portrayed. Thanks to social media, women now have additional avenues for business and networking. In recent years, particularly during the time of covid-19, there has been a noticeable increase in the number of women entrepreneurs working on social media platforms in Bangladesh [10]. Women get more liberty, freedom, empowerment, independence, and autonomy due to social media and their online presence on social media. It empowers women to experiment and discover new things. Social media expands individuals' opportunities for job creation, self-employment, and financial security, ultimately contributing to the economy. As such, social media boosts women's confidence and economic standing as entrepreneurs, benefiting their personal growth, society, and progress. The findings of this study are supported by previous studies [44].

However, women's empowerment is critical for a society to develop into a knowledge-based society in which women's progress positively impacts individual families, ultimately benefiting the community and country. Social media amplifies women's voices, which have been suppressed and repressed for years [24]. Not only women social media also connects individuals and amplifies their voices. Their participation in online business contributes to family income. Thus, H2 is supported through this study, indicating the social media entrepreneurship of rural women affects family income contribution. Overall, the study's conclusions are supported by the variables, and the evidence gathered from the statistical analysis suggests that promoting social media entrepreneurship among rural women can be an effective strategy for empowering them and promoting sustainable development.

## 6 Conclusions, policy implications, and future study scope

The study examines the effect of rural women's participation in social media entrepreneurship on family income contribution and sustainable development. The study employs PLS-SEM techniques to analyze 376 responses collected from clothing business entrepreneurs on the online platform. After analyzing the measurement and structural model and checking the model fitness, the study finds significant relationships between social media entrepreneurship and family income, family income and sustainable development, and social media entrepreneurship and sustainable development. This study provides strong evidence for the positive impact of social media entrepreneurship on sustainable development in Bangladesh. Specifically, the results support all three hypotheses, indicating that there is a significant positive relationship between women's participation in social media entrepreneurship and rural women's family income, as well as a significant positive relationship between women's participation in social media entrepreneurship and sustainable development, and between rural women's family income and sustainable development.

Women, particularly in developing nations, lack empowerment in most sectors. They typically depend on male family members, yet education and society's attitude toward women is changing [45]. Social networking is one of the most effective instruments for empowering women. The ability to make decisions about the household, economics, healthcare, and childcare and involvement in political and social activities all contribute to raising awareness about the need to empower women in society. Bangladesh's situation is rapidly changing. Through new media, particularly social media, women are becoming more empowered in decision-making, earning, and involvement in socio-economic, cultural, and political activities.

The findings of this study have important theoretical implications for understanding the relationship between women's participation in social media entrepreneurship and sustainable development. Firstly, this study provides empirical evidence that women's participation in social media entrepreneurship positively impacts sustainable development in Bangladesh. This supports the idea that women's economic empowerment and gender equality are essential components of sustainable development, as recognized by the feminist theory of evolution. Secondly, this study highlights the potential of social media entrepreneurship as a tool for empowering women and promoting sustainable development. By providing rural women in Bangladesh with access to new economic opportunities and markets, social media entrepreneurship has the potential to increase their financial contributions and improve their standard of living. Finally, this study also finds that rural women's family income contribution positively affects sustainable development in Bangladesh. This highlights the importance of ensuring that women's economic contributions are valued and recognized and that policies are in place to support women's access to resources and opportunities. The study's results suggest that women's economic empowerment and participation in social media entrepreneurship can be powerful tools for promoting sustainable development and that policies and programs that support women's empowerment should be prioritized.

Over the past two decades, Bangladesh has made substantial strides in enhancing the well-being of women and girls. With women constituting more than half of the country's population, their active involvement in socio-economic spheres has experienced a remarkable upswing [19]. Bangladesh stands as a beacon of women's empowerment and progress, setting an exemplary precedent through its dedicated efforts in this domain. The nation is currently undergoing a profound societal transformation, propelled by its steadfast commitment to fostering women's advancement. Notably, the workforce now accommodates a robust number of women, totaling 18.6 million in 2017, a noteworthy increase from 16.2 million in 2010. Bangladesh's standing as the 50th nation among 153 in the 2020 Global Gender Gap Report underscores its commendable achievements. In stark contrast, Nepal, Sri Lanka, India, and Pakistan trail behind, occupying ranks of 101, 102, 112, and 151, respectively. This positioning not only underscores Bangladesh's impressive progress but also highlights the significant challenges still faced by neighboring nations in achieving gender parity and women's empowerment.

This study has at least four practical implications: Firstly, encouragement for women's participation in social media entrepreneurship: The positive effect of the involvement of women in social media entrepreneurship on sustainable development suggests that policymakers and development organizations should prioritize and invest in programs and initiatives that support women's participation in social media entrepreneurship. This could include providing training, technical assistance, and access to finance for rural women in Bangladesh to start and grow their online clothing businesses. Secondly, the importance of family income contribution: The study's finding that rural women's family income contribution positively affects sustainable development in Bangladesh highlights the importance of valuing and recognizing women's economic contributions. Policies and programs should be implemented to support women's access to resources and opportunities and ensure that women's contributions to their families and communities are valued and rewarded. Thirdly, empowerment of rural women: By highlighting the positive impact of social media entrepreneurship on rural women's income and sustainable development in Bangladesh, this study can be used to encourage and motivate rural women to participate in social media entrepreneurship and to support their efforts to grow their businesses and improve their economic prospects. Finally, the importance of access to ICT: This study also highlights the importance of access to information and communication technologies (ICT) in promoting sustainable development and empowering women. Policies and programs should be implemented to ensure that rural women in Bangladesh can access ICT, including the internet and digital devices, to participate in social media entrepreneurship and other economic opportunities. Overall, the findings of this study have practical implications for policymakers, development organizations, and individuals working to promote sustainable development and gender equality in Bangladesh. By highlighting the positive impact of women's participation in social media entrepreneurship and the importance of family income contribution, this study can inform the development of policies and programs that support women's empowerment and promote sustainable development.

The study has the following contributions: Firstly, the structural model shows that a positive effect of women's participation in social media entrepreneurship on sustainable development exists within online clothing shops in Bangladesh. Secondly, through ICT project offerings, Bangladesh can create employment opportunities for rural women. Consistently this study also proves social media entrepreneurship increases women's contribution to their family income. Furthermore, this study finds that rural women's family income contribution positively affects sustainable development in Bangladesh. Thirdly, the methodological contribution made use of structural equation modelling using the PLS approach, which assesses the model's fitness with regards to the reliability and validity of each examined construct and the overall model. The results of this study, then, are based on the most appropriate model, and they add a new dimension of methodology to the literature [18,23,24,66,70].

The study has some limitations. First, the study uses a few indicators to develop the constructs like family income, social media entrepreneurship, and sustainable development. Future studies may include more indicators and constructs to develop the structural model. Second, the study is the cross-sectional type that lacks the groups of online businesses. The future research may include another construct named networking of women entrepreneurs to check the association and the degree to which networking of women entrepreneurs helps achieve sustainable development. Because networking of women entrepreneurs helps spread the positive impacts of women's empowerment, they feel they can contribute to economic development with panel data to generalize the study. Finally, the study did not consider the open opinion of the entrepreneurs. Future research may include both open-ended and closed-ended questions for entrepreneurs.

## Author contribution statement

Md. Mominur Rahman: Conceived and designed the experiments; Performed the experiments; Analyzed and interpreted the data; Wrote the paper.

Bishawjit Chandra Deb, PhD: Conceived and designed the experiments.

Muhammad Shajib Rahman; Mohammad Jubair Hossain; Md. Jahid Hasan: Contributed reagents, materials, analysis tools or data.

## Data availability statement

Data will be made available on request.

## Additional information

No additional information is available for this paper.

## Declaration of competing interest

The author declares that there is no competing interest.

## Abbreviations

SEM: Structural equation modelling, DV: Discriminant validity, AVE: Average variance extracted, CR: Composite reliability, TLI: Tucker–Lewis's index, RMSEA: Root mean square error approximation, SRMR: Standardized root mean square residual, RWP: Rural Women's Participation, FIN: Family Income contribution, SD: Sustainable development, PLS: Partial Least Square.
